# Socioeconomic Status, Sex, and Obesity in a Large National Cohort of 15–87-Year-Old Open University Students in Thailand

**DOI:** 10.2188/jea.JE20090014

**Published:** 2010-01-05

**Authors:** Sam-ang Seubsman, Lynette L-Y. Lim, Cathy Banwell, Nintita Sripaiboonkit, Matthew Kelly, Christopher Bain, Adrian C. Sleigh

**Affiliations:** 1 Thai Health-Risk Transition Project, School of Human Ecology, Sukhothai Thammathirat Open University, Nonthaburi, Thailand; 2 National Centre for Epidemiology and Population Health, The Australian National University, Canberra, Australia; 3 Cancer Registry, Medical Informatics, Ramathibodhi Hospital, Mahidol University, Bangkok, Thailand; 4 The University of Queensland, School of Population Health, Brisbane, Australia

**Keywords:** body mass index, weight, obesity, socioeconomic status, Thailand

## Abstract

**Background:**

As obesity increases, middle-income countries are undergoing a health-risk transition. We examine the association between socioeconomic status (SES) and emerging obesity in Thailand, and ascertain if an inverse relationship between SES and obesity has appeared.

**Methods:**

The data derived from 87 134 individuals (54% female; median age, 29 years) in a national cohort of distance-learning Open University students aged 15–87 years and living throughout Thailand. We calculated adjusted odds ratios for associations of SES with obesity (body mass index, ≥25) across 3 age groups by sex, after controlling for marital status, age, and urbanization.

**Results:**

Obesity increased with age and was more prevalent among males than females (22.7% vs 9.9%); more females were underweight (21.8% vs 6.2%). Annual income was 2000 to 3000 US dollars for most participants. High SES, defined by education, income, household assets, and housing type, associated strongly with obesity—positively for males and inversely for females—especially for participants younger than 40 years. The OR for obesity associated with income was as high as 1.54 for males and as low as 0.68 for females (*P* for trend <0.001).

**Conclusions:**

Our national Thai cohort has passed a tipping point and assumed a pattern seen in developed countries, ie, an inverse association between SES and obesity in females. We expect the overall population of Thailand to follow this pattern, as education spreads and incomes rise. A public health problem of underweight females could emerge. Recognition of these patterns is important for programs combating obesity. Many middle income countries are undergoing similar transitions.

## INTRODUCTION

Worldwide, obesity has shown contrasting socioeconomic associations in 144 studies reported from 1933 to 1988. In developed countries, affluent groups—especially affluent women—are less likely to be obese; in contrast, in developing countries a positive association between obesity and socioeconomic status (SES) is common.^1^ An inverse association between SES and obesity was confirmed in developed countries,^2^ in all US states,^3^ and in other studies conducted from 1980 through 2002.

Jeffery proposed that the obesity risk for those with low SES in developed countries reflects both economic discrimination and economic deprivation: obese persons are restricted in social advancement and the poor resort to cheap high-calorie foods and spend less on recreational exercise.^4^ In contrast, in developing countries the poor have restricted food intakes and do manual work, whereas the rich have more food resources and labor-saving devices.^4^ In addition, given the association in developing countries between poverty and thinness, greater wealth and higher SES are often displayed by being fat.^1^

But how do developing countries transition from the traditional direct association between SES and obesity? Unfortunately, information on this topic is limited. To gather the necessary data from countries in transition, and to devise suitable interventions for such settings, it is important to study obesity as it emerges. The existence of an income threshold at which obesity in developing countries “tips” from a direct to an inverse relationship with SES has been suggested, and evidence indicates that this threshold may be lower for women. This tipping point may due, in part, to the fact that opportunities for occupational exercise decline at a certain level of economic development, which may initially affect poor urban women when average incomes reach approximately US$2500 per capita.^5^ It could also reflect the differential effects of education for men and women, as women convert health and nutritional knowledge into behavioral change earlier than their male counterparts.

In the last few decades, economic development in Southeast Asia has changed diets and ways of life,^6^ leading to falling birth and death rates and emerging obesity.^7^ For example, in Thailand from 1983 to 2006, sugar consumption nearly tripled, from 12.7 to 33.2 kg per person per year.^8^ The Thai diet now includes more oil, fats, and meat, and less vegetables and fruit.^9^^,^^10^ Over the same period, in addition to obesity, chronic diseases such as cancer, hypertension, and diabetes have become increasingly prevalent.^11^

This study investigates the relationship between SES and obesity in Thailand. We use baseline data from a large national Thai cohort study (TCS) begun in 2005. Our results for proximate lifestyle risk factors—notably diet and physical activity—have already been published.^12^ Here, we focus on SES, so as to identify indirect influences on nutrition and the ongoing obesity transition. We report the differential effects of increasing SES on males and females in different age groups, noting any evidence of an income tipping point for the SES–obesity relationship.

## METHODS

### Data

The Thai Health-Risk Transition: A National Cohort Study is an ongoing population-based study of distance-learning university students residing throughout Thailand. This cohort comprised students enrolled at Sukhothai Thammathirat Open University (STOU). It is referred to as an open university because it does not require high school graduates to pass an entrance test. The baseline 2005 TCS data reported here include information on most of the socioeconomic, cultural, and lifestyle questions considered pertinent to the obesity problem, along with measures of health outcomes. Details on population selection and methodology have been reported.^11^ Briefly, the 2005 student register listed about 200 000 names and addresses: a 20-page questionnaire was mailed to each student and 87 134 (44%) responded; 54% were females and the median age was 29 years. Data scanning, verification, and correction were then conducted using Scandevet, a program developed by a research team from Khon Kaen University. Further data editing was completed using SQL and SPSS software, and for epidemiological analysis we used SPSS and Stata. A total of 85 886 people submitted data on sex and body mass index (BMI). For any given analysis, individuals with missing information were excluded; therefore, the totals presented in the analysis may differ slightly.

### Variables and categories

Height in centimeters (cm) and weight in kilograms (kg) were self-reported and measured without shoes. BMI was derived from the ratio of a person’s weight divided by the square of the height in meters and recorded in kg/m^2^. Information on other self-reported details chosen for this analysis included potential modifiers or confounders of SES–obesity associations (age, sex, marital status, urban or rural residence in 2005 and when aged 10–12 years), as well as SES indicators (highest level of education, monthly personal income, household assets, and housing type).

We used Asian cutoffs to classify adults as underweight (BMI <18.5), normal (18.5 to <23), overweight (23 to 24.9), or obese (≥25).^12^^–^^15^ Obesity was classified using a cutoff of BMI ≥25 because other studies have provided information on health risk outcomes for Asian populations at this level.^14^ Age groups were divided into 3 bands: a younger group (15–25 years), a middle age group (26–39 years), and an older age group (40 years and older). Marital status was classified as single or partnered. Self-reported urban or rural residence in 2005, and when aged 10–12 years, was used to create 4 life-course urbanization categories: rural to rural (RR), rural to urban (RU), urban to rural (UR), and urban to urban (UU).

There is no standard socioeconomic indicator in Thailand, so we defined SES by educational attainment, monthly personal income, household assets classified by replacement values, and housing type. These variables enabled us to study a range of SES indicators, not just income or education. Cohort members were asked to report their highest educational attainment before enrollment at STOU, which was classified into 3 categories: high school or lower, vocational school diploma or the equivalent, or university education. Personal income was reported in Thai baht (approximate exchange rate in 2005, 40 Baht = US$1). Respondents indicated their monthly baht incomes by choosing from 5 categories: ≤3000, 3001–7000, 7001–10 000, 10 001–20 000, or 20 000 or higher. Household assets included general domestic items—microwave oven, electric fan, air conditioner, computer, radio, video/vcd recorder, washing machine, water heater, and telephone. Replacement values in baht were then calculated and categorized into 3 groups: low (≤30 000), middle (30 001–60 000), and high (>60 000). Type of housing was classified as detached, semi-detached, townhouse, shop-front house, or apartment.

### Statistical analysis

Overall mean BMIs and the prevalences of underweight, overweight, and obesity, with 95% confidence intervals, were calculated for males and females. Differences between means and proportions were tested by analysis of variance and the chi-square test, respectively. All *P* values were 2-tailed and the significance level was set at 5%. For each sex, unadjusted obesity prevalences and 95% confidence intervals were calculated for each categorical value, for all variables analyzed.

Males and females were separately stratified into 3 age groups, and the odds ratio (OR) association of an obesity outcome with each SES indicator, ie, education, personal income, household assets, and housing type, was assessed by logistic regression models. Important confounding factors—age as a continuous variable, marital status, and life-course urbanization—were included in each SES model, because each was shown on bivariate analysis to be significantly associated with obesity and with various measures of SES. ORs for obesity used the lowest SES category as the reference. Tests for trend were performed using SES indicators as ordinal variables. Interactions between sex and each SES indicator’s effect on obesity were revealed by separately comparing the results obtained for males and females. Age cohort effects were assessed by comparing SES–obesity associations for the 3 age groups. Because the statistical interactions of SES with sex and with age cohort in the adjusted SES–obesity models were highly significant (*P* < 0.001), we present the results separately for the 2 sexes and the 3 age groups.

### Ethical considerations

Informed written consent was obtained from all participants, and ethics approval was obtained from the Sukhothai Thammathirat Open University Research Committee and the Australian National University Human Research Ethics Committee.

## RESULTS

Table 1 summarizes the BMI and other attributes of the STOU cohort. The mean BMI was significantly higher in males (22.9) than in females (20.9). The prevalence of underweight was much higher in females than males (21.8% vs 6.2%), but more males were overweight (21.7% vs 9.7%) and obese (22.7% vs 9.9%). All these differences in weight category prevalences were statistically significant (*P* < 0.001).

**Table 1.** Body mass index, demographic attributes, and socioeconomic status (SES) in a cohort of 85 886 Thai distance-learning students

	Males			Females			Difference
	
	*n*	Mean (SD)	%	*n*	Mean (SD)	%	
Weight status							
Body mass index (BMI)	38 825	22.9 (3.3)		47 061	20.9 (3.2)		<0.0001

Weight classification (%)							
Underweight (BMI <18.5)	2391		6.2	10 268		21.8	<0.0001
Normal (18.5 ≤ BMI < 23)	19 204		49.5	27 567		58.6	
Overweight (23 ≤ BMI < 25)	8413		21.7	4563		9.7	
Obese (BMI ≥25)	8817		22.7	4663		9.9	

Demographic attributes							
Age, y	39 478	32.2 (8.8)		47 636	29.1 (7.5)		<0.0001
Age group (%)							
15–25	10 036		25.4	19 029		39.9	<0.001
26–39	21 432		54.3	23 519		49.4	
≥40	8010		20.3	5088		10.7	
Marital Status							
Single	18 765		49.0	29 078		62.9	<0.001
Married/living with a partner	19 567		51.0	17 160		37.1	
Urbanization Status							
Rural-Rural	17 609		45.3	20 127		42.7	<0.001
Rural-Urban	12 616		32.4	14 812		31.4	
Urban-Rural	1699		4.4	2007		4.3	
Urban-Urban	6977		17.9	10 170		21.6	

Socioeconomic status							
Education level							
High school	21 748		55.2	20 699		43.6	<0.001
Vocational school	8902		22.6	14 560		30.6	
University	8734		22.2	12 247		25.8	
Personal income, baht/month^a^							
<7000	13 612		35.4	22 019		47.4	<0.001
7001–10 000	8821		22.9	10 973		23.6	
10 001–20 000	10 920		28.4	9643		20.8	
20 001+	5147		13.4	3804		8.2	
Household assets, baht^b^							
<30 000	15 917		40.5	19 263		40.4	<0.001
30 001–60 000	12 328		31.4	14 267		29.9	
>60 000	11 018		28.1	13 883		29.1	
Housing type							
Detached	24 920		63.7	30 221		63.9	<0.001
Semi-detached	1174		3.0	1182		2.5	
Shop-front house	3716		9.5	4493		9.5	
Townhouse	3247		8.3	4540		9.6	
Apartment	4695		12.0	5628		11.9	
Other	1643		4.2	1513		3.2	

^a^US$1 = 42 Thai baht at the time of the survey (2005).

^b^Replacement value.

On average, women had moderately higher educational attainment and men had substantially higher personal income (Table 1). Approximately one-third of males and half of females reported incomes less than 7000 baht per month (<US$2100 per year). The values of reported household assets were similar for males and females: less than 30 000 baht (<US$750) for 40% and greater than 60 000 baht (>US$1500) for approximately 29%. The reported housing types were similar for males and females.

As compared to the youngest age band, obesity rates doubled for both sexes in the middle age band (26–39 years), and roughly doubled again for those aged 40 years or older (Table 2). Single males and females had much lower rates of obesity than those who were partnered. For both sexes, the rate of obesity among those urbanized by the age of 12 years was substantially higher than among those who had been rural dwellers at the same age. The prevalence of obesity tended to rise in males as educational level increased, but changed only slightly in females. The prevalence of obesity increased with rising income in both sexes. As the value of assets rose, obesity prevalence increased substantially in both sexes. Obesity prevalence and housing type showed similar patterns in males and females: respondents who resided in a townhouse were the most likely to be obese; those who lived in an apartment were substantially less likely to be obese.

**Table 2.** Obesity prevalence by sex and demographic and socioeconomic status (SES) in a cohort of 85 886 Thai distance-learning students

	Males (*n* = 38 825)			Females (*n* = 47 061)		
	
	No. obese	Prevalence %	95% CI	No. obese	Prevalence %	95% CI
Overall study population	8817	22.7	22.3–23.1	4663	9.9	9.6–10.2
Age group, y						
15–25	1032	10.4	9.8–11.0	1101	5.9	5.5–6.2
26–39	4847	23.0	22.4–23.5	2468	10.6	10.3–10.8
≥40	2938	37.8	36.7–38.9	1094	22.0	20.9–23.2
Marital status						
Single	5767	15.4	14.8–15.9	2386	8.3	8.0–8.6
Partnered	2842	30.0	29.4–36.7	2153	12.7	12.2–13.2
Urbanization status						
Rural-Rural	3402	19.6	19.1–20.2	1716	8.6	8.2–9.0
Rural-Urban	2780	22.4	21.7–23.2	1163	7.9	7.5–8.4
Urban-Rural	471	28.2	26.1–30.4	305	15.4	13.8–17.0
Urban-Urban	2034	29.5	28.4–30.6	1422	14.2	13.5–14.8
Education level						
High school	4515	21.3	20.7–21.8	2081	10.2	9.7–10.6
Vocational school	1963	22.3	21.5–23.2	1328	9.2	8.8–9.7
University	2298	26.7	25.3–27.6	1239	10.2	9.7–10.8
Personal Income, baht						
≤7000	2021	15.2	14.6–15.8	1825	8.4	8.0–8.7
7001–10 000	1688	19.4	18.5–20.2	892	8.2	7.7–8.7
10 001–20 000	3076	28.7	27.8–29.6	1248	13.2	12.5–13.8
>20 000	1874	37.2	35.9–38.5	588	15.8	14.6–17.0
Household assets, baht^a^						
<30 000	2554	16.3	15.8–16.9	1453	7.6	7.3–8.0
30 001–60 000	2793	23.0	22.3–23.8	1447	10.3	9.8–10.8
>60 000	3426	31.6	30.7–32.5	1747	12.7	12.1–13.3
Housing type						
Detached house	5277	21.5	21.0–22.1	2877	9.6	9.3–10.0
Semi-detached house	287	24.7	22.2–27.2	120	10.3	8.6–12.1
Shop-front house	1030	28.0	26.6–29.5	523	11.7	10.8–12.7
Townhouse	944	29.6	28.0–31.2	559	12.5	11.5–13.4
Apartment	922	19.9	18.7–21.0	420	7.6	6.9–8.2

^a^Replacement value.

The adjusted urbanization–obesity odds ratios (controlled for age and marital status; data not shown) revealed a similar pattern to the crude obesity prevalence analysis in Table 2: urban residence at age 12 years was a substantial risk factor for subsequent adult obesity in both sexes, but odds ratios attenuated with age and were insignificant in the older groups. In contrast to urbanization, the SES associations with obesity differed considerably by both sex and age group, after adjusting for the influence of age, marital status, and life-course urbanization (Tables 3 and 4). Overall, higher educational attainment was associated with a higher rate of obesity in males and a lower rate of obesity in females, although this trend was more obvious in participants younger than 40 years. When analyses were stratified to enable comparison of patterns for urban and rural groups, the effects of SES on obesity continued to result in opposing trends for males and females, regardless of place of residence. Thus, urbanization is a confounder, not a modifier, of sex-specific SES effects on obesity.

**Table 3.** Associations between indicators of socioeconomic status (SES) and obesity by age group among 38 825 males in a cohort of Thai distance-learning students

	Crude ORs, all ages, 95% CI		Adjusted ORs^a^, 95% CI					

			15–25 yrs		26–39 yrs		≥40 yrs	
Education level								
High school	1		1		1		1	
Vocational school	1.06	1.00–1.13	1.06	0.90–1.25	1.09	1.00–1.19	1.07	0.95–1.22
University	1.35	1.27–1.43	1.27	1.05–1.54	1.15	1.06–1.25	1.01	0.90–1.13
(*P*-trend)			(0.021)		(<0.001)		(0.760)	

Personal income, baht/month								
≤7000	1		1		1		1	
7001–10 000	1.34	1.25–1.44	0.92	0.78–1.10	1.15	1.04–1.26	1.11	0.90–1.37
10 001–20 000	2.25	2.11–2.40	1.13	0.89–1.44	1.27	1.15–1.39	1.30	1.10–1.53
>20 000	3.31	3.08–3.57	1.05	0.63–1.73	1.54	1.35–1.75	1.34	1.13–1.58
(*P*-trend)			(0.686)		(<0.001)		(<0.001)	

Household assets, baht								
<30 000	1		1		1		1	
30 001–60 000	1.53	1.44–1.63	1.44	1.23–1.69	1.14	1.05–1.24	1.24	1.08–1.43
>60 000	2.36	2.23–2.51	2.00	1.67–2.39	1.45	1.33–1.58	1.43	1.25–1.64
(*P*-trend)			(<0.001)		(<0.001)		(<0.001)	

Housing type^b^								
Apartment	1		1		1		1	
Semi-detached/shop/townhouse	1.57	1.44–1.72	1.71	1.29–2.26	1.19	1.06–1.34	1.25	1.01–1.54
Detached	1.10	1.02–1.19	1.39	1.07–1.79	1.07	0.96–1.19	1.09	0.89–1.34

^a^Adjusted ORs were from a logistic regression model for each SES factor, ie, age (years), urbanization, and marital status.

^b^There was no natural order for housing type, so *P*-trend was not calculated.

**Table 4.** Associations between indicators of socioeconomic status (SES) and obesity by age group among 47 061 females in a cohort of Thai distance-learning students

	Crude ORs, all ages (95% CI)		Adjusted ORs^a^, (95% CI)					

			15–25 yrs		26–39 yrs		≥40 yrs	
Education level								
High school	1		1		1		1	
Vocational school	0.89	0.84–0.97	0.82	0.70–0.95	0.94	0.84–1.04	1.01	0.85–1.21
University	1.00	0.94–1.08	0.80	0.67–0.97	0.80	0.72–0.89	0.81	0.68–0.95
(*P*-trend)			(0.006)		(<0.001)		(0.021)	

Personal income, baht/month								
≤7000	1		1		1		1	
7001–10 000	0.98	0.89–1.05	0.68	0.57–0.81	0.81	0.72–0.91	1.14	0.86–1.50
10 001–20 000	1.66	1.54–1.79	0.88	0.67–1.14	0.87	0.78–0.97	1.09	0.88–1.36
>20 000	2.05	1.86–2.27	0.79	0.43–1.43	0.68	0.57–0.81	1.00	0.80–1.25
(*P*-trend)			(0.004)		(<0.001)		(0.737)	

Household assets, baht								
<30 000	1		1		1		1	
30 001–60 000	1.38	1.28–1.49	1.18	1.01–1.37	0.97	0.87–1.08	1.18	0.94–1.47
>60 000	1.76	1.63–1.89	1.40	1.18–1.64	0.96	0.86–1.08	0.92	0.75–1.14
(*P*-trend)			(<0.001)		(0.502)		(0.096)	

Housing type^b^								
Apartment	1		1		1		1	
Semi-detached/shop/townhouse	1.64	1.46–1.85	1.48	1.15–1.91	1.15	0.98–1.35	0.96	0.70–1.31
Detached	1.29	1.16–1.44	1.06	0.83–1.34	1.16	0.99–1.35	1.08	0.80–1.46

^a^Adjusted ORs were from a logistic regression model for each SES factor, ie, age (years), urbanization, and marital status.

^b^There was no natural order for housing type, so *P*-trend was not calculated.

The adjusted ORs for the effect of income also differed by sex. For males in the middle and older age groups, there was a monotonic trend of increasing obesity with increasing income; for females in the younger and middle age groups, the odds of obesity fell substantially with rising income. However, there was little effect due to income among older women. The pattern for the value of household assets also differed by sex, with a strongly positive monotonic effect for males in all age groups and a weaker, partially inverted, pattern for females. Regarding the effect of housing type, males living in semi-detached, shop-front houses, or townhouses were at highest risk in all age groups, but the risk decreased with age. Among females, the risk pattern was similar, but weaker, and was not evident among older women.

## DISCUSSION

In this large cohort of distance-learning Open University students, aged 15 to 87 years and living throughout Thailand, obesity was strongly associated with age, sex, and socioeconomic status. The age effect was strong for both sexes: obesity rose with age. A sex effect was also prominent—obesity rates were much higher in males than in females. The overall prevalences of overweight and obesity in males were more than double the levels noted in females; however, underweight was present in nearly 22% of females and only 6% of males. Obesity was also strongly positively associated with living with a partner and with urban residence during childhood.

In many of the present analyses, participants aged 40 years or older had different patterns to the 2 younger age cohorts, suggesting that younger Thais over the last 2 or 3 decades have been differentially exposed to upstream factors that influence obesity, such as life-course urbanization and higher socioeconomic status. It should be noted that the 40 and older age group included 248 people aged over 60 years; however, this small number of age outliers did not distort any of the calculated proportions or associations, as the results were nearly identical when those aged over 60 years were excluded.

The effects of SES interacted with sex for all 4 indicators. Sex differences were most evident for personal SES indicators with natural ordering—education and income; the association was direct among males and inverse among females for both of these indicators. The 2 other SES indicators—value of household assets and housing type—were probably more related to family status than to personal attributes. Both value of household assets and housing type (semi-detached, shop-front house, or townhouse) showed strong positive associations with obesity in males, and weaker, mixed, associations in females. On Kendall’s tau-c test, ordered categories for household assets were substantially more concordant with ordered income categories for males than for females. Because men, on average, earn more than women, the value of household assets (and housing type) would be more likely to reflect their personal income. Among females, asset value and housing type may correspond more closely with family income. Furthermore, it should be noted that housing type was not clearly associated with SES; for example, a detached house might be associated with either income extreme. Also, 15- to 25-year-olds often showed a pattern of association that differed in the level of significance and even in direction from those of older age groups, possibly because younger students depend more on the resources of their families.

We did not analyze energy intake and physical activity, which are important determinants of obesity. For this cohort, data on these proximate factors have been reported separately: the main variables (and their population attributable fractions for obesity) were sedentariness (2.3%), more than 4 hours per day of screen time with television and computers (10.7%), and frequent consumption of fried foods (18.5%).^12^ These variables are likely to mediate in the causal pathway from SES to obesity, and thus were not considered confounders in the present analysis.

A feature of this study was that self-reporting was used to gather information from the cohort. This method of data collection gave us access to Thais living and working throughout the country, and, along with the capacity for follow up by personal contact, was designed (and pre-tested) to enhance data reliability.^11^ In addition, the large size of the cohort generated excellent statistical power, even after controlling for covariates during analysis. This self-reporting may be a limitation for the study, however, as it allows for potential errors to arise in the data, due to the nature of self-reporting, eg, incorrect self-reporting of weight or height. To explore the possibility of such errors, in 2004 and 2007 we measured the height and weight of 741 STOU students visiting the main campus of the university and compared the results to self-reported values. Correction of the present results based on those findings would produce a 5% increase in the prevalence of obesity in males and a 2% increase in females, which would be unlikely to alter the SES associations reported here.^12^ In addition, others have noted that self-reported height and weight are sufficiently accurate for the purposes of population analyses of obesity.^16^

Respondents, who were identifiable for follow up, may have limited their disclosure of personal information such as partner status, occupation, and income. However, study leaders assured the respondents of the confidentiality of the study, and almost no respondents left such items unanswered. We are therefore confident of the overall integrity of the data. Finally, we note that the 44% response rate to the baseline questionnaire might indicate the presence of a variety of biases in the data. Although we are not able to determine the actual biases, the geographic distribution, sex, age, and employment profiles of our respondents differed little from those reported for the student body as a whole by STOU.

Another issue arising from the study design is the focus on distance-learning Open University students, which allowed us to take advantage of an excellent postal contact system and enabled us to sample from the whole adult Thai population. STOU students are not wealthier than average Thais and are unable to leave work or their families to study. By utilizing them, we were able to capture much of the extant variation in life histories, current living circumstances, and health status in the Thai population. We have found that they represented well the geographic regions of Thailand, as well as a wide range of other factors.^11^ To cite one example, a substantial proportion of our cohort, 35% of males and 47% of females, had quite low incomes (<7000 baht per month, or US$5.50 per day). However, the focus on distance-learning university students does limit our ability to represent the educational profile of the general Thai population, among whom the majority have much lower levels of education. Even so, as much of the health-risk transition underway in middle-income countries is mediated by education^17^^–^^19^; we believe that our cohort is ahead of national education trends and thus sheds light on dynamics that will eventually spread to the whole population.^11^^,^^20^

Historically, many studies noting an association between SES and obesity in developing countries have shown that high socioeconomic status was positively related to obesity in men, women, and children.^1^ However, since 1982, an increasing number of reports have extended these observations to middle-income countries, especially those where annual per capita incomes exceed US$2500.^5^ In such settings, an inverse relationship between SES and obesity may develop among women, but not men, which is similar to observations made in developed countries. In Thailand, per capita income first exceeded US$2500 in 1995, decreased below that level in 1997, and then rebounded above it from 2003.^21^ Two Thai obesity studies over that period are relevant. The first analyzed the National Health Examination Survey from 1997 (NHES II) and concluded that obesity was associated with female sex, marital partnering, and increasing age, but not with education or occupation.^22^ The second report re-examined the 1997 data and also included NHES III data obtained in 2004^23^; analyses stratified by sex showed a tendency to an inverse association between education and obesity for females, and the opposite for males, especially in the 25–<30 BMI category. Singapore (which exceeded the US$2500 mark in 1975) is an example of such an SES–BMI transition in a developed Asian country. A study conducted there in the late 1990s found that females with a high SES (based on income, education, and housing) had a significantly lower adjusted odds of obesity—a pattern that was not found in men.^24^ In Korea, a similar pattern was found when income and education were used as SES indicators.^25^

The present study confirms that among a large national sample of Thai distance-learning university students, most with modest incomes in the range of 2000 to 3000 US dollars, the inverse association between SES and obesity that is characteristic of developed countries is already present in females, especially among those younger than 40 years. Moreover, our sample is better educated than the general Thai population and females studying at STOU had remarkably lower overall rates of obesity than those reported by Aekplakorn using the NHES III data that had been collected only 1 year before.^23^ In our sample, after correcting for self-reporting, the rate of obesity (BMI, 25+) was 28% for males and 12% for females; the NHES III rates were 24% for males and 35% for females. Therefore, STOU males were a little more obese than their national counterparts, but STOU females had an obesity prevalence that was only about one-third the national average.

We conclude that our study has identified an ongoing transition in the effect of SES on BMI. The high rate of underweight among STOU women (21.8% vs 6.2% for men) suggests that educated Thai women are growing more concerned about their weight. This may reflect concerns about social attractiveness, which have been observed in developed countries.^4^ Thai television is already saturated with weight reduction advertisements directed at females, and Thai female celebrities are almost always thin. We expect that this sex-modified pattern of obesity will spread rapidly throughout Thailand as education improves, and that a new national problem—underweight—may emerge for young Thai females. Thus, the indirect and proximate forces driving the emerging obesity trend in Thailand (urbanization, changing diets, physical inactivity, and, for men, increasing education and income) will be countered by rising education and income for females. These competing influences need to be considered in the implementation of interventions to combat a national problem that already warrants much public health concern.
